# Designing with living systems in the synthetic yeast project

**DOI:** 10.1038/s41467-018-05332-z

**Published:** 2018-07-27

**Authors:** Erika Szymanski, Jane Calvert

**Affiliations:** Science, Technology and Innovation Studies, University of Edinburgh, Old Surgeons’ Hall, High School Yards, Edinburgh, EH1 1LZ UK

## Abstract

Synthetic biology is challenged by the complexity and the unpredictability of living systems. While one response to this complexity involves simplifying cells to create more fully specified systems, another approach utilizes directed evolution, releasing some control and using unpredictable change to achieve design goals. Here we discuss SCRaMbLE, employed in the synthetic yeast project, as an example of synthetic biology design through working with living systems. SCRaMbLE is a designed tool without being a design tool, harnessing the activities of the yeast rather than relying entirely on scientists’ deliberate choices. We suggest that directed evolution at the level of the whole organism allows scientists and microorganisms to “collaborate” to achieve design goals, suggesting new directions for synthetic biology.

## Abstract (web summary)

Synthetic biology often views the organism as a chassis into which a circuit can be inserted. Here the authors explore the idea of the organism as a core aspect of design, aiding researchers in navigating the genetic space opened up by SCRaMbLE.

## Introduction

Synthetic biology is often seen as a collection of diverse approaches to designing and building with DNA^1^. One way of unifying synthetic biology as a field is to emphasize its orientation toward improving scientific control over increasingly well-characterized biological systems^2,3^. While synthetic biologists have achieved notable success in increasing control and improving characterization, attempts to systematically engineer biology are often seen to be impeded by the complexity and unpredictability of biological systems. Pointing to this central difficulty, commentators have suggested that a solution may lie in simplifying host cells to create more reliable and better specified systems for further engineering work^4^. We use the example of the SCRaMbLE system developed in the *Saccharomyces cerevisiae* 2.0 (Sc2.0) project to illustrate an alternate vision, in which bio-engineers let go of some control to work with living organisms to achieve design goals.

In his 2005 review of synthetic biology, Drew Endy listed “an inability to avoid or manage biological complexity” first among “challenges that greatly limit the engineering of biology”^5^. This has become a refrain across the field, with the complexity characteristic of all living cells given as a fundamental impediment to what has been called the “bottom-up” design of artificial cells^6^. The complex contextual dependency of biological parts is described as both a driver of the need for standards and a challenge in implementing them^7,8^. And biological complexity is cited as a reason for the gap between the ever-increasing capacities of DNA synthesis and the limited capabilities of functional DNA assembly^9,10^. Amidst these challenges, observers have noted that in practice, many successful synthetic biology projects do not appear to involve engineering because they rely too much on random chance and too little on standardized practices in ways “incompatible with canonical engineering”^11–13^; for some, nevertheless, achieving “‘true’ engineering” remains a viable goal^14^.

In seeking this vision of engineering biological systems, extensive work has aimed to maximize control over DNA sequence and assembly, genetic pathway function, and cell behavior. Notwithstanding the field’s many accomplishments, achieving desired levels of control has been difficult^15,16^. Biological systems remain complex and unpredictable. Fully specified rational engineering remains challenging and rare. While successful synthetic biology projects may be narrated as stories fitting engineering-style design-build-test cycles, they can also be described as tales of chance or trial and error. In a prominent example, the J. Craig Venter Institute team involved in constructing the smallest-yet genome able to support life reported that deliberately designing and engineering a minimal genome from existing knowledge of microbial functioning failed to yield a viable organism. Trial and error succeeded, however, where rational design did not^17^.

Aims to make biological systems more controllable, and practical difficulties in doing so, point to central tensions in synthetic biology involving how living organisms and machines must be handled differently, how far the “machine concept of the organism” is taken^18^, and what is done when organisms do not behave like machines. The goal to create so-called living machines remains central to much synthetic biology, and indeed the field’s flagship student event is called the international Genetically Engineered Machine (iGEM) competition. Simultaneously, however, evidence continues to point to ways in which microorganisms are distinctly non machine-like. Noise (in the sense of unwanted signal disturbance), for example, is typically something to be avoided or eliminated when engineering machines, yet, in biological systems noise is not only inevitable but beneficial for population survival^19,20^. Variation is a liability in groups of presumably identical machines, but a resource in evolving populations^21^. Modularity of form and modularity of function are linked in many engineered machines, but frequently are not linked in living organisms^12^. Promoters and other seemingly basic biological parts do not reliably perform identical functions when employed in different contexts^22^.

Engineering to maximize the controllability of a biological machine is, however, not the only mode of operation in synthetic biology, nor is creating standardized biological parts a universal goal^1,16,23^. Directed evolution strategies are increasingly employed to harness biological complexity and unpredictably as assets rather than attempting to erase them as liabilities^24^. Some reviews have presented directed evolution and other randomness-leveraging strategies as temporary or imperfect solutions to the problem of having insufficient knowledge to permit rational engineering, a problem which their authors expect synthetic biologists to solve in the future^12,25,26^. However, we interpret these strategies differently: as creating possibilities for working with living systems rather than trying to exert total control over them, wherein the goals of harnessing biology for particular purposes take precedence over the goals of transforming biology into an engineering discipline. We suggest that directed evolution operating at the level of the whole organism offers a potential trajectory for synthetic biology that deviates from the aim of making increasingly controllable biological machines, instead enabling scientists and microorganisms to “collaborate” to achieve design goals.

In this perspective we use the SCRaMbLE (Synthetic Chromosome Rearrangement and Modification by LoxPsym-mediated Evolution) system developed in the Sc2.0 project to illustrate our reasons for making this suggestion. SCRaMbLE, as a directed evolution strategy, typically requires yeast cells to adapt to challenging environments by devising genomic solutions that scientists would have been very unlikely to devise through rational engineering. Rather than attempting to erase the unique features of the yeast to make it more like a reliable engineering material, SCRaMbLE relies on the yeast’s genomic flexibility and capacity for homologous recombination, taking advantage of unique characteristics of the organism while accepting less directed scientific control. We first briefly explain the biological structure and function of SCRaMbLE, then we examine how SCRaMbLE shapes the role of the microorganism central to the project and argue that it enables us to see synthetic biology in a different light, as a process of designing with living systems.

## SCRaMbLEing synthetic yeast

Sc2.0 is an internationally distributed project aiming to comprehensively redesign and synthesize the first complete synthetic eukaryotic genome. The Sc2.0 genome sequence was initially specified in silico, beginning with an existing *S. cerevisiae* genome derived from the common laboratory strain SC288C and incorporating multiple genome-wide modifications. Construction of one or more of the sixteen chromosomes has then been taken up by eleven consortium-member laboratories^25,27^. The first completed synthetic chromosome was announced in *Nature* in 2014^28^; five additional completed chromosomes were announced in a March 2017 special issue of *Science*^29–33^. Amongst numerous other changes designed to increase the robustness and flexibility of Sc2.0 without decreasing its fitness^24^, SCRaMbLE tends to generate the most excitement.

SCRaMbLE leverages the well-established Cre-lox system employed as a tool for biological investigation since the 1980s^34^. loxP recognition sites for the Cre recombinase enzyme, originally directional, have been re-engineered to permit unbiased bidirectional recombination^35^. The Sc2.0 genome sequence includes these loxPsym sites as inserts in the 3’ untranslated region of all genes annotated as “non-essential” in the Saccharomyces Genome Database (SGD) and at some additional significant chromosomal landmarks. loxPsym sites are recognition sequences for Cre recombinase, which is therefore able to catalyze large-scale genomic rearrangements including insertions, deletions, duplications, and inversions between any two loxPsym sites within or across synthetic chromosomes. Cre recombinase is introduced fused to the murine estrogen binding domain (EBD) such that Cre-EBD is only translocated to the nucleus when bound to estradiol. SCRaMbLE activity can therefore be turned on or off, up or down by introducing or withdrawing estradiol or by inducing cells to lose the plasmid on which Cre-EBD is carried.

Unsurprisingly, the vast majority of induced rearrangements appear to be lethal. However, a small percentage of SCRaMbLEd genomes are able to sustain cell growth and division. SCRaMbLE was initially conceived as a means to evaluate the lethality of simultaneously inactivating multiple genes in combination, a longstanding problem in yeast genetics as the number of individual experiments necessitated by increasing the number of simultaneous knock-outs increases factorially^28^. SCRaMbLE allows the rapid evaluation of potentially infinite combinatorial gene inactivations and enables the identification of cells which survive on an increasingly smaller complement of functional genes, working toward one or more “minimal genomes” for *S. cerevisiae* and addressing other questions in yeast genetics and evolutionary biology^28,36^.

As “an inducible evolution system”^36^ to create population-level diversity in yeast strains bearing one or more synthetic chromosomes, the system also has applications in directed evolution to optimize whole-cell phenotypes^7^. In this sense, SCRaMbLE can be seen as a response to a perceived need in synthetic biology for combinatorial construction strategies which, paired with appropriate screening tools, can identify optimal designs in the absence of sufficient systems-level knowledge to rationally design such assemblies^9^. While the complete synthetic genome remains under construction, SCRaMbLE is being explored as a means to generate strains adapted for growth under industrially relevant conditions.

As is typical for directed evolution experiments, SCRaMbLE experiments involve an appropriately prepared heterogeneous cell population—or in this case, a population with capacity to generate heterogeneity—and a selective environment. Genomic heterogeneity is introduced via Cre recombinase activity in yeast cells bearing at least one synthetic chromosome or part of a synthetic chromosome and the Cre-EBD plasmid. This population is exposed to selective growth conditions over multiple generations, either in a chemostat or via batch transfer. Cells with genetic variations enabling survival—or, ideally, better-than-wild-type growth—under the chosen conditions can be identified by assaying the growth rate of the population and screening individual clones when population growth rate peaks. Atypically for directed evolution experiments, SCRaMbLE creates potentially large and extensive genetic rearrangements, making the explorable experimental space unusually expansive compared with random mutagenesis or other natural sources of genomic novelty^21^. SCRaMbLE has been demonstrated to generate large rearrangements highly unlikely to be seen in nature^37^.

SCRaMbLE would appear to be randomness under scientific control, in that the system can be turned on and off at will, but SCRaMbLE is both less random and less controlled than this brief description might suggest. The sequences of SCRaMbLEd genomes will only ever be semi-random. Not all rearrangements are possible and not all possible rearrangements are equally likely: many will prove lethal and therefore never be seen, and steric constraints influence which pairs of loxPsym sites are most likely to be proximal. Moreover, the range of potential rearrangements is constrained by initial decisions about where to locate the loxPsym sites. And the SCRaMbLE process itself is not perfectly controllable. Small amounts of Cre recombinase may be translocated to the nucleus even in the absence of estradiol so the surest way to halt SCRaMbLE activity is to induce cells to lose the Cre-EBD plasmid. Eliminating the plasmid from cells identified as having rearranged genomes of potential interest requires at least one generation of growth, however, such that those genotypes may have been lost to further SCRaMbLE activity before they can be assayed.

While SCRaMbLE may be a designed tool, therefore, it is not precisely a design tool in the sense that it does not help bring about the physical instantiation of a conceptually designed object^13^. SCRaMbLE works toward a preconceived effect or outcome—that is, toward a cell able to grow under specified conditions—but it does not make it possible to control the structure of the physical construction—the genome sequence—which satisfies that outcome. Indeed, the opposite is true: the scientist controls the growth conditions, but gives up control over the genome sequence. SCRaMbLE is therefore an example of a different mode of working in synthetic biology wherein the goal remains designing and building with DNA, but the scientist is not solely in control of the designing.

## Yeast as design tool and engineering material

SCRaMbLE relies on several unique capacities of yeast as an engineering material. Though the vast majority of rearrangements kill the cell, all of what might be considered failures—that is, rearrangements unable to sustain life—disappear because cells that do not grow become invisible to analysis. Consequently, SCRaMbLE relies on large populations of cells being easy to generate, and on both those populations and individual cells being disposable. Many different kinds of culturable cells have these properties, even if all engineering materials do not. However, SCRaMbLE takes advantage of several particular characteristics of *S. cerevisiae*. For example, yeast are “highly amenable to and tolerant of genetic manipulation”^38,39^, since the yeast genome is relatively loosely organized with few intrachromosomal contacts^40,41^. The SCRaMbLE system may not work as well in a genome more highly and densely regulated by interregional contacts, as are most bacterial genomes.

## Rethinking the role of the microorganism

SCRaMbLE provokes us to ask: what is the role of the yeast? While this question might seem unreasonably anthropomorphic, we find it productive to adopt this perspective and think of the specific capabilities of the yeast as central to the operation of SCRaMbLE and to the success of the Sc2.0 project as a whole. Applying terms developed for use in the human-scale world to microorganisms is invariably flawed, and yeast surely act in ways not perfectly coherent with frames for understanding macro-organismal behavior. Our interest is in finding empirically supportable ways of discussing yeast participation that enable making this participation a visible part of the research process, and therefore something that can be accounted for, incorporated in, and valued as part of scientific work.

SCRaMbLE not only makes yeast a particular kind of engineering material by reshaping the yeast genome in the image of its anticipated future uses, but also by employing the yeast in multiple roles (summarized in Table 1). The yeast cell is a convenient container for a molecule of interest, an experimental system for combinatorial chemistry experiments with genomic DNA. Individual cells isolate and contain individual experiments to generate large numbers of semi-randomly varied molecules in a search for new compounds with specific properties^38,42,43^. The yeast cell is a DNA synthesis factory in the sense that it produces these molecules of interest. Having produced these molecules, the yeast cell is also a screening tool. Whether or not the cell grows is the first step in measuring the success of genomic rearrangements and identifying molecules for additional analysis.

**Table 1:** Multiple roles yeast can play in SCRaMbLE experiments

**Possible role of the yeast**	**Associated function**	**Example**
Container	Cell provides a compartment for combinatorial DNA experiments	Varied DNA assemblies in individual cells are able to be assessed independently
Screening tool	Cell provides initial evaluation for success of DNA assemblies	Yeast cell growth/absence of growth indicates presence/absence of desired construct
User	Cell provides feedback about functionality of DNA assemblies	Yeast cell growth/growth rate indicates appropriate functionality of construct

We could, in this sense, say that the yeast cell is the primary user of the DNA molecule. Although conceiving of the yeast as the user of synthetic DNA may be unconventional, we think that it is productive in that it becomes possible to ask new questions about relationships between “designers” and “users.” If microorganisms are thought of as users of synthetic DNA, how would synthetic biologists practice user-centered design strategies? We are not making an essentialist argument about whether yeast (or any other organism, for that matter) is or is not capable of deliberate collaboration; instead, we suggest that recognizing that yeast can be seen as an active participant can be a useful perspective for expanding our understanding of synthetic biology.

SCRaMbLE is by no means unique in these respects, as using cells as containers and screening tools is conventional across biotechnology. The yeast cell must be able to successfully employ the information contained within a rearranged DNA molecule to support cellular function, whether that molecule is a SCRaMbLEd chromosome or a rationally designed sequence. If it cannot, irrespective of how coherent the DNA sequence is with any rational logic of the scientist, the SCRaMbLEd DNA molecule cannot be successfully used for its most important purpose, the cell fails to thrive, and the experiment fails.

In some forms of participatory design, users take on equal status with designers as co-creators of new technologies built through a collaborative process of identifying needs, goals, and strategies. In this light, SCRaMbLE experiments could be seen as participatory design processes in which a technology—the SCRaMbLEd genome—is the subject of a negotiation between designer and user: the scientists give the yeast a design(ed) tool (SCRaMbLE) and a goal (e.g., growing at stress-inducing temperatures), and the yeast respond by using their resources and the SCRaMbLE tool to achieve the goal. SCRaMbLE experiments may not be very good participatory design exercises in that information is exchanged between scientist and yeast primarily or exclusively at the beginning and the end of the experiment, unless the scientist communicates by altering the growth conditions or the yeast respond by dying, growing very slowly, or otherwise signaling their non-participation in achieving the goal. With that in mind, it is interesting to speculate about what a more engaged participatory design process would entail in experimental terms.

In a very material sense, however, the yeast are already collaborators or coworkers in SCRaMbLE experiments, and in Sc2.0 more broadly. Yeast are responsible for substantial portions of the work involved in synthesizing the Sc2.0 genome. Via homologous recombination and technologies that rely on homologous recombination^38,44^, yeast assemble chemically synthesized or bacterially amplified DNA into increasingly large segments of synthetic chromosomes. Human scientists do not yet know how to do homologous recombination, but yeast are so proficient at DNA assembly that assembly “in yeasto”^38^ remains a recourse for assemblies that prove difficult via Gibson, Golden Gate, or other means. Moreover, the design of SCRaMbLE experiments incorporates yeast “decision-making.” SCRaMbLE maintains many simultaneous possible trajectories within an unusually large experimental space, then allows the yeast to participate in “choosing” which of these potential trajectories to continue following.

Whether these contributions make yeast a tool or a collaborator is largely about the assumptions made about what the yeast is in the first place—a passive engineerable chassis made active only through the intervention of an intentional user, or an active contributor to a design process. The line between tool and collaborator, however, is neither fixed nor black-and-white. Collaborators sometimes do nothing without being directed by another intentional force. Tools shape work employing those tools in ways not always fully controlled or intended by the user. Tools are sometimes addressed as collaborators (“please, computer, just run this one little program for me”) and collaborators are sometimes treated like tools.

Rather than making a single decision amongst any one of these frames, when yeast almost certainly participate in multiple frames simultaneously, a better question might be what kinds of relationships are desirable or productive to have with yeast. More important than bringing yeast into the picture of doing synthetic biology in any particular role, perhaps, is that SCRaMbLE and other whole-organism-directed evolution strategies bring the yeast into the picture at all. This is not a point about *S. cerevisiae* specifically, though it could be argued that yeast has sometimes been overlooked in synthetic biology in preference for working with *E. coli*; rather, all organisms often seem absent from conversations that tend to focus on genes and pathways. SCRaMbLE makes the organism visible in both conceptual and material ways. This visibility itself makes it possible to imagine a trajectory for synthetic biology that involves designing with biological systems rather than aiming to simplify, constrain, and completely control them.

## Designing with biological systems

Kant defined “an organized product of nature [as] that in which everything is an end and reciprocally a means”^45^. An alternate definition of a living organism comes from NASA: “a self-sustaining chemical system capable of Darwinian evolution”^46^. By both definitions, genetic parts construction and even much whole genome-focused synthetic biology work is about designing and building in organisms rather than designing and building organisms themselves. Indeed, design paradigms that conceptualize biological systems as passive platforms or chassis and attempt to modify those systems to increase their passivity would seem to curtail their biological qualities for the purpose of building on top of them. Even protocell-building projects focused on creating simplified organisms capable of Darwinian evolution are arguably building with non-living materials in advance of constructing something which is alive, rather than dealing with evolution and other markers of life as features of the material being engineered^47^.

SCRaMbLE, in contrast, appears to be a strategy for designing and building with living biological systems that not only accounts for but also depends on the peculiar biological qualities of the system. Yeast cells work with SCRaMbLE and selective pressures to optimize genomes along different lines than scientists would take to rationally achieve the same goals. The way yeast cells optimize genomes using SCRaMbLE is also not the same way yeast cells would optimize genomes without SCRaMbLE. A SCRaMbLE experiment can, therefore, be thought of as a human-yeast collaboration in which the scientist designs a tool and the yeast uses that tool to create something that meets the scientist’s goals, but which the scientist would not have predicted and would not have been able to create alone.

SCRaMbLE is contiguous with other directed evolution strategies in the sense that it involves designing something that will evolve toward the desired product, but differs in focusing on the whole cell rather than on specific enzymes or pathways of interest. Directed evolution strategies are often used in synthetic biology contexts to account for the complex operation of a whole cell while optimizing a protein, pathway, or network within that cell^26,48^. Whole-organism-directed evolution strategies such as SCRaMbLE, in contrast, emphasize working with the organism rather than working toward the molecule. In this sense, these strategies may signal a return of the organism in a field in which the organism has often been invisible in technical discussions about engineering biological systems.

While SCRaMbLE is a new system enabling new kinds of experiments and design strategies, it leverages long-standing genetic techniques. The system is also oriented toward achieving a particular functional outcome rather than toward making the process cohere with a particular engineering process, creating standardized parts or circuits, or employing a design-build-test cycle. SCRaMbLE could be read, then, as indicative of a shift away from trying to establish synthetic biology as an engineering process and toward trying to achieve functional biological products independent of whether the process of doing so matches the engineering principles that characterized early hopes of the field.

## SCRaMbLEing toward shifts in synthetic biology

As social scientists investigating synthetic biology’s aspirations to apply engineering principles to biological systems, SCRaMbLE appears to us to signify three shifts. Together, these form the basis for our hypothesis that a new trajectory for synthetic biology is emerging, de-emphasizing control in ways that make space for working more consciously with living systems. The first is an attention to the biology in biological engineering. The second is a growing attitude toward capacities for randomness and heterogeneity as assets of working with biological systems. The third is a change in the vision of engineering mobilized in engineering biology.

SCRaMbLE encourages us to think of the organism as the context for engineering and is, in this sense, akin to other whole cell approaches to directed evolution. Unlike most of these other approaches, however, SCRaMbLE does not employ the whole cell as a means to accommodate cellular context in the process of engineering a molecule or pathway of interest, but aims to employ an engineering approach with the whole cell itself. In SCRaMbLE experiments, the organism can be seen both as the desired product and as an important contributor to the process of arriving at that product. Relying on specific characteristics of the yeast, SCRaMbLE demonstrates a mode of allowing for and even harnessing biological complexity rather than attempting to engineer biological complexity out of the system.

Biological engineering, as embraced in synthetic biology, has largely been about the application of engineering principles to biological systems. Nevertheless, how amenable biology is to being engineered, and what might be meant by engineering in biological contexts, remain open questions. Synthetic biological routes to making biology engineerable often remove or suppress responses of the host cell to engineering interventions. In contrast, SCRaMbLE relies upon the activity of the organism: in generating genomic diversity, accommodating drastic recombinations, and screening for genomes capable of sustaining growth and that might be suitable for additional applications.

Employing randomness as a design tool challenges how engineering is typically imagined in synthetic biology. Visions of engineering emphasizing rational design and complete control are often manifested in synthetic biology to distinguish between mere tinkering and “real engineering”—where “real engineering” is interpreted as involving wholly predictable processes devoid of randomness—and to invoke a future in which biological engineers do the latter. This vision reflects only part of the work done by engineers of non-biological systems—engineers who often do describe their work in terms of tinkering, trial and error, and serendipity^49^. Such visions also essentially assume one amongst multiple possible, as yet untested futures for biological knowledge. Statements that look forward to the future of synthetic biology routinely envision that increases in biological knowledge will permit the manipulation of biological systems in these predictable ways.

At least three important assumptions are embedded in those visions. First, the assumption is that biological systems can be described by first principles following the same pattern of similar principles once unknown, but now at the core of modern chemistry and physics. Second, the assumption is that biological knowledge will move toward—and, one hopes, eventually reach—defining those principles, as has happened and continues to happen in chemistry and physics. Third, the assumption is that knowing these principles will enable manipulating biological systems in more systematic ways patterned after the ways engineers work with non-living systems. But what if biological knowledge does not move in this direction, and does not find biological systems to be fundamentally united by a set of fixed principles? What if increases in biological knowledge make biological systems less engineerable, not more so? Identifying “fundamental organizing principles” has been described as the “intellectual front end” of systems biology, but evidence that such principles exist remains scarce^50^ Kant’s philosophy of science assumes that all nature is governed by laws, but he contrasts the laws of physics against living organisms, which are driven by a holistic sense of purpose rather than by reductive first principles^51^.

## Conclusions

In 2008, O’Malley et al. argued that “the fate of synthetic biology hinges on its capacity to deal with the complex properties of highly variable biological systems” and that “if synthetic biology’s future is to be more than a modest contributor to “analytic” biology, it needs to develop broader engineering principles that do more than mimic those of non-biological engineering”^1^. One way of thinking about those engineering principles is that, through increasing knowledge about evolution and other biological processes, evolution could become central to new engineering paradigms^52^. SCRaMbLE suggests an additional route: the work of devising these new paradigms might be shared with the microorganisms themselves.

## References

[1] O'Malley MA, Powell A, Davies JF, Calvert J (2008). Knowledge-making distinctions in synthetic biology. BioEssays.

[2] Balmer AS, Bulpin KJ (2013). Left to their own devices: post-ELSI, ethical equipment and the International Genetically Engineered Machine (iGEM) competition. BioSocieties.

[3] Schyfter P, Frow E, Calvert J (2013). Guest editorial: synthetic biology: making biology into an engineering discipline. Eng. Stud..

[4] Xavier JC, Patil KR, Rocha I (2014). Systems biology perspectives on minimal and simpler cells. Microbiol. Mol. Biol. Rev. MMBR.

[5] Endy D (2005). Foundations for engineering biology. Nature.

[6] Walde P (2010). Building artificial cells and protocell models: experimental approaches with lipid vesicles. BioEssays News Rev. Mol. Cell. Dev. Biol..

[7] Cobb RE, Sun N, Zhao H (2013). Directed evolution as a powerful synthetic biology tool. Methods San. Diego Calif..

[8] Casini A, Storch M, Baldwin GS, Ellis T (2015). Bricks and blueprints: methods and standards for DNA assembly. Nat. Rev. Mol. Cell Biol..

[9] Dietz S, Panke S (2010). Microbial systems engineering: First successes and the way ahead. BioEssays.

[10] Davidsohn N (2015). Accurate predictions of genetic circuit behavior from part characterization and modular composition. ACS Synth. Biol..

[11] Porcar M, Peretó J (2012). Are we doing synthetic biology?. Syst. Synth. Biol..

[12] Porcar M, Danchin A, de Lorenzo V (2015). Confidence, tolerance, and allowance in biological engineering: the nuts and bolts of living things. BioEssays.

[13] Kogge W, Richter M (2013). Synthetic biology and its alternatives. Descartes, Kant and the idea of engineering biological machines. Stud. Hist. Philos. Sci. Part C. Stud. Hist. Philos. Biol. Biomed. Sci..

[14] Heinemann M, Panke S (2006). Synthetic biology—putting engineering into biology. Bioinformatics.

[15] Kwok R (2010). Five hard truths for synthetic biology. Nat. News.

[16] Porcar M (2016). Synthetic biology: from having fun to jumping the gun. NanoEthics.

[17] Service RF (2016). Synthetic microbe has fewest genes, but many mysteries. Science.

[18] Nicholson DJ (2013). Organisms ≠ machines. Stud. Hist. Philos. Sci. Part C. Stud. Hist. Philos. Biol. Biomed. Sci..

[19] Knuuttila T, Loettgers A (2014). Varieties of noise: analogical reasoning in synthetic biology. Stud. Hist. Philos. Sci. Part A.

[20] Purnick PEM, Weiss R (2009). The second wave of synthetic biology: from modules to systems. Nat. Rev. Mol. Cell Biol..

[21] Rothschild LJ (2010). A powerful toolkit for synthetic biology: over 3.8 billion years of evolution. BioEssays.

[22] Carr SB, Beal J, Densmore DM (2017). Reducing DNA context dependence in bacterial promoters. PLoS One..

[23] Finlay SC (2013). Engineering biology? Exploring rhetoric, practice, constraints and collaborations within a synthetic biology research centre. Eng. Stud..

[24] Sauro HM (2014). Synthetic biology: how best to build a cell. Nature.

[25] Richardson SM (2017). Design of a synthetic yeast genome. Science.

[26] Cobb RE, Chao R, Zhao H (2013). Directed evolution: past, present, and future. AIChE J..

[27] Jovicevic D, Blount BA, Ellis T (2014). Total synthesis of a eukaryotic chromosome: redesigning and SCRaMbLE-ing yeast. BioEssays News Rev. Mol. Cell. Dev. Biol..

[28] Annaluru N (2014). Total synthesis of a functional designer eukaryotic chromosome. Science.

[29] Xie ZX (2017). “Perfect” designer chromosome V and behavior of a ring derivative. Science.

[30] Wu Y (2017). Bug mapping and fitness testing of chemically synthesized chromosome X. Science.

[31] Mitchell LA (2017). Synthesis, debugging, and effects of synthetic chromosome consolidation: synVI and beyond. Science.

[32] Zhang W (2017). Engineering the ribosomal DNA in a megabase synthetic chromosome. Science.

[33] Shen Y (2017). Deep functional analysis of synII, a 770-kilobase synthetic yeast chromosome. Science.

[34] Sauer B (1987). Functional expression of the cre-lox site-specific recombination system in the yeast Saccharomyces cerevisiae. Mol. Cell. Biol..

[35] Lindstrom DL, Gottschling DE (2009). The mother enrichment program: a genetic system for facile replicative life span analysis in Saccharomyces cerevisiae. Genetics.

[36] Dymond J, Boeke J (2012). The Saccharomyces cerevisiae SCRaMbLE system and genome minimization. Bioeng. Bugs.

[37] Shen Y (2015). SCRaMbLE generates designed combinatorial stochastic diversity in synthetic chromosomes. Genome Res. Gr..

[38] Mitchell LA (2015). Versatile genetic assembly system (VEGAS) to assemble pathways for expression in S. cerevisiae. Nucleic Acids Res..

[39] Maxmen, A. Synthetic yeast chromosomes help probe mysteries of evolution. *Scientific American* https://www.scientificamerican.com/article/synthetic-yeast-chromosomes-help-probe-mysteries-of-evolution/. Accessed: 31st July 2017 (2017). 10.1038/nature.2017.21615 28300123

[40] Mercy G (2017). 3D organization of synthetic and scrambled chromosomes. Science.

[41] Olson MV (1991). 1 Genome structure and organization in Saccharomyces cerevisiae. Cold Spring Harb. Monogr. Arch..

[42] Guo Y (2015). YeastFab: the design and construction of standard biological parts for metabolic engineering in Saccharomyces cerevisiae. Nucleic Acids Res..

[43] Jensen ED (2017). Transcriptional reprogramming in yeast using dCas9 and combinatorial gRNA strategies. Microb. Cell Factor..

[44] Chandran S, Shapland E (2017). Efficient assembly of DNA using yeast homologous recombination (YHR). *Methods Mol*. Biol. Clifton NJ.

[45] Kant I, Guyer P, Matthews E (2000). Critique of the Power of Judgment.

[46] Benner SA (2010). Defining life. Astrobiology.

[47] Szostak JW, Bartel DP, Luisi PL (2001). Synthesizing life. Nature.

[48] Bassalo MC, Liu R, Gill RT (2016). Directed evolution and synthetic biology applications to microbial systems. Curr. Opin. Biotechnol..

[49] Schyfter P (2013). How a ‘drive to make’ shapes synthetic biology. Stud. Hist. Philos. Sci. Part C. Stud. Hist. Philos. Biol. Biomed. Sci..

[50] Dhar, P. K. & Giuliani, A. Laws of biology: why so few? *Syst. Synth. Biol.* **4**, 7–13 (2010). 10.1007/s11693-009-9049-0 PMC2816229 20186254

[51] Breitenbach, A. in *Kant and the Laws of Nature* (eds Michela, M. & Breitenbach, A.) 237–255 (Cambridge University Press, Cambridge, 2017).

[52] Ginsberg AD, Calvert J, Schyfter P, Elfick A, Endy D (2014). Synthetic Aesthetics: Investigating Synthetic Biology’s Designs on Nature.

